# Correction: Specialty detection in the context of telemedicine in a highly imbalanced multi-class distribution

**DOI:** 10.1371/journal.pone.0296113

**Published:** 2023-12-14

**Authors:** Alaa Alomari, Hossam Faris, Pedro A. Castillo

An additional affiliation is missing for the second author. Hossam Faris is also affiliated with the Information Technology Department, King Abdullah II School for Information Technology, The University of Jordan, Jordan.
